# Acquired resistance to combination treatment through loss of synergy with MEK and PI3K inhibitors in colorectal cancer

**DOI:** 10.18632/oncotarget.8692

**Published:** 2016-04-11

**Authors:** Bhaskar Bhattacharya, Sarah Hong Hui Low, Mei Ling Chong, Dilys Chia, King Xin Koh, Nur Sabrina Sapari, Stanley Kaye, Huynh Hung, Touati Benoukraf, Richie Soong

**Affiliations:** ^1^ Cancer Science Institute of Singapore, National University of Singapore, Singapore; ^2^ Department of Pharmacy, National University of Singapore, Singapore; ^3^ Department of Pathology, National University of Singapore, Singapore; ^4^ Drug Development Unit, Royal Marsden NHS Trust, The Royal Marsden NHS Foundation Trust, London, United Kingdom; ^5^ Laboratory of Molecular Endocrinology, National Cancer Centre of Singapore, Singapore

**Keywords:** acquired resistance, combination therapy, MEK inhibitors, PI3K inhibitors, synergy

## Abstract

Historically, understanding of acquired resistance (AQR) to combination treatment has been based on knowledge of resistance to its component agents. To test whether an altered drug interaction could be an additional factor in AQR to combination treatment, models of AQR to combination and single agent MEK and PI3K inhibitor treatment were generated. Combination indices indicated combination treatment of PI3K and MEK inhibitors remained synergistic in cells with AQR to single agent but not combination AQR cells. Differences were also observed between the models in cellular phenotypes, pathway signaling and drug cross-resistance. Genomics implicated *TGFB2-EDN1* overexpression as candidate determinants in models of AQR to combination treatment. Supplementation of endothelin in parental cells converted synergism to antagonism. Silencing of *TGFB2* or *EDN1* in cells with AQR conferred synergy between PI3K and MEK inhibitor. These results highlight that AQR to combination treatment may develop through alternative mechanisms to those of single agent treatment, including a change in drug interaction.

## INTRODUCTION

Combination therapy, entailing treatment using two or more agents together, has become a common strategy for optimizing treatment efficacy, and reducing toxicity and drug resistance [1]. The strategy is based on the principle that an additional effect is generated by combining agents over using them individually. These effects can be synergistic, additive, or antagonistic, namely greater than, equal to, or worse than the added effects of the component agents respectively.

Drug resistance remains a major cause of treatment failure [2]. The resistance can be due to intrinsic factors that manifest in a lack of initial drug activity. Resistance can also be acquired, developing as a result of changes induced by the selection pressure of drug treatment. Many mechanisms of acquired resistance (AQR) have been identified, including changes in drug efflux, metabolism and detoxification, and acquired DNA mutations, gene amplification, pathway redundancy, crosstalk and feedback, and the enrichment of resistant subclones [3].

In recent times, the emergence of molecular-targeted therapy has led to an increased consideration of combination therapy as a means of circumventing drug resistance. However, AQR to combination therapy can still be expected, with evidence readily available from the clinic. What is unclear is whether mechanisms of AQR to combination therapy are the same as those for monotherapy, and whether an altered drug interaction could also be a factor. In this study, models of AQR to the prominent combination of MEK and PI3K inhibitors [4–9] were generated concurrently with models to single agent treatment. Selective loss of synergy in combination treatment was observed in the cells with AQR to combination treatment but not single agent treatment, presenting loss of synergy as a novel mechanism of AQR to be considered in the application of combination therapy.

## RESULTS

### Generation of AQR and loss of synergy

HCT116 cells with *KRAS* G13D and *PIK3CA* H1047R mutations (cancer.sanger.ac.uk) were cultured in the presence of both AZD6244 (MEK inhibitor) and BKM120 (PI3K inhibitor) at IC_50_ concentrations of each agent, AZD6244 alone (2 treatments of ½ IC50 concentrations), BKM120 alone (2 treatments of ½ IC_50_ concentrations), or vehicle (2 treatments of 0.25% DMSO). Two treatments were provided for all models to minimize bias from the number of treatments of the cells.

After prolonged treatment, HCT116 cells cultured with both AZD6244 and BKM120 became resistant to combination AZD6244 and BKM120 treatment (designated as “HCT116CR” cells) compared to HCT116 cells cultured with DMSO (“HCT116DM” cells) (Table 1). Combination index (CI) analysis [10] indicated that AZD6244 and BKM120 were antagonistic in HCT116CR cells, while they were synergistic in HCT116DM cells. HCT116CR cells also displayed increased resistance to single agent treatment with AZD6244, but not BKM120.

**Table 1:** IC_50_ and combination index values of treatment with various drugs and their combinations in HCT116-derived cells

Cell Line	HCT116DM	HCT116AR	HCT116BR	HCT116CR
AZD6244 IC_50_ (μM)	2.8 ± 0.03	30.2 ± 0.12*	10.2 ± 0.20*	6.1 ± 0.31*
BKM120 IC_50_ (μM)	1.3 ± 0.01	4.8 ± 0.04*	5.2 ± 0.19*	1.2 ± 0.02
AZD6244 + BKM120 IC_50_ (μM)	0.8 ± 0.02	0.9 ± 0.04	0.9 ± 0.02	10 ± 2.34*
AZD6244 + BKM120 CI_fu0.5_	0.15 ± 0.031	0.29 ± 0.028	0.21 ± 0.007	1.98 ± 0.210*
GDC0973 IC_50_ (μM)	5.7 ± 0.81	20.3 ± 2.32*	22.1 ± 1.94*	17.8 ± 1.66*
BYL719 IC_50_ (μM)	9.8 ± 1.20	30.2 ± 3.11*	38.2 ± 4.04*	25.6 ± 2.85*
GDC0973 + BYL719 IC_50_ (μM)	0.5 ± 0.05	0.5 ± 0.02	0.6 ± 0.05	10.9 ± 1.81*
GDC0973 + BYL719 CI_fu0.5_	0.16 ± 0.083	0.28 ± 0.042	0.39 ± 0.012	1.96 ± 0.381*

The IC_50_ values of AZD6244, BKM120, GDC0973, and BYL719 as single agents and in combination (in the presence of the other drug at fixed ratio of their IC_50_ values) are indicated. CI values for fraction unaffected at IC_50_ (fu0.5) are also given. Additivity = 1, Antagonism > 1, Synergy < 1.

* *p* < 0.05 for differences in IC_50_ values compared to HCT116DM, and for differences to 1 for CI values.

HCT116 cells treated with AZD6244 alone (“HCT116AR” cells) and BKM120 alone (“HCT116BR” cells) displayed AQR to their respective treatments. Cross-resistance was observed for HCT116AR cells to BKM120, as well as for HCT116BR cells to AZD6244. Nonetheless, the combination of AZD6244 and BKM120 remained synergistic in HCT116AR and HCT116BR cells.

To confirm that the AQR and loss of synergy was not compound specific, the sensitivity of the cells to GDC0973 (MEK inhibitor) and BYL719 (PI3K inhibitor) treatment was assessed. Similar patterns of AQR, cross-resistance and loss of synergy was observed with these agents in respective cells (Table 1). The only difference in pattern was an increased resistance of HCT116CR cells to BYL719.

To confirm that the observations were not specific to HCT116 cells, LoVo (*KRAS* G13D mutant, cancer.sanger.ac.uk) colorectal cancer cells with AQR to AZD6244 (“LoVoAR”), BKM120 (“LoVoBR”) and their combination (“LoVoCR”) were generated using the same methods applied to HCT116 cells. The cells exhibited similar patterns of resistance to AZD6244 and BKM120 treatment, as well as GCD0973 and BYL719 treatment, as observed for HCT116 cells (Supplementary Table S1).

### Pathway signaling and inhibition

**Figure 1:**
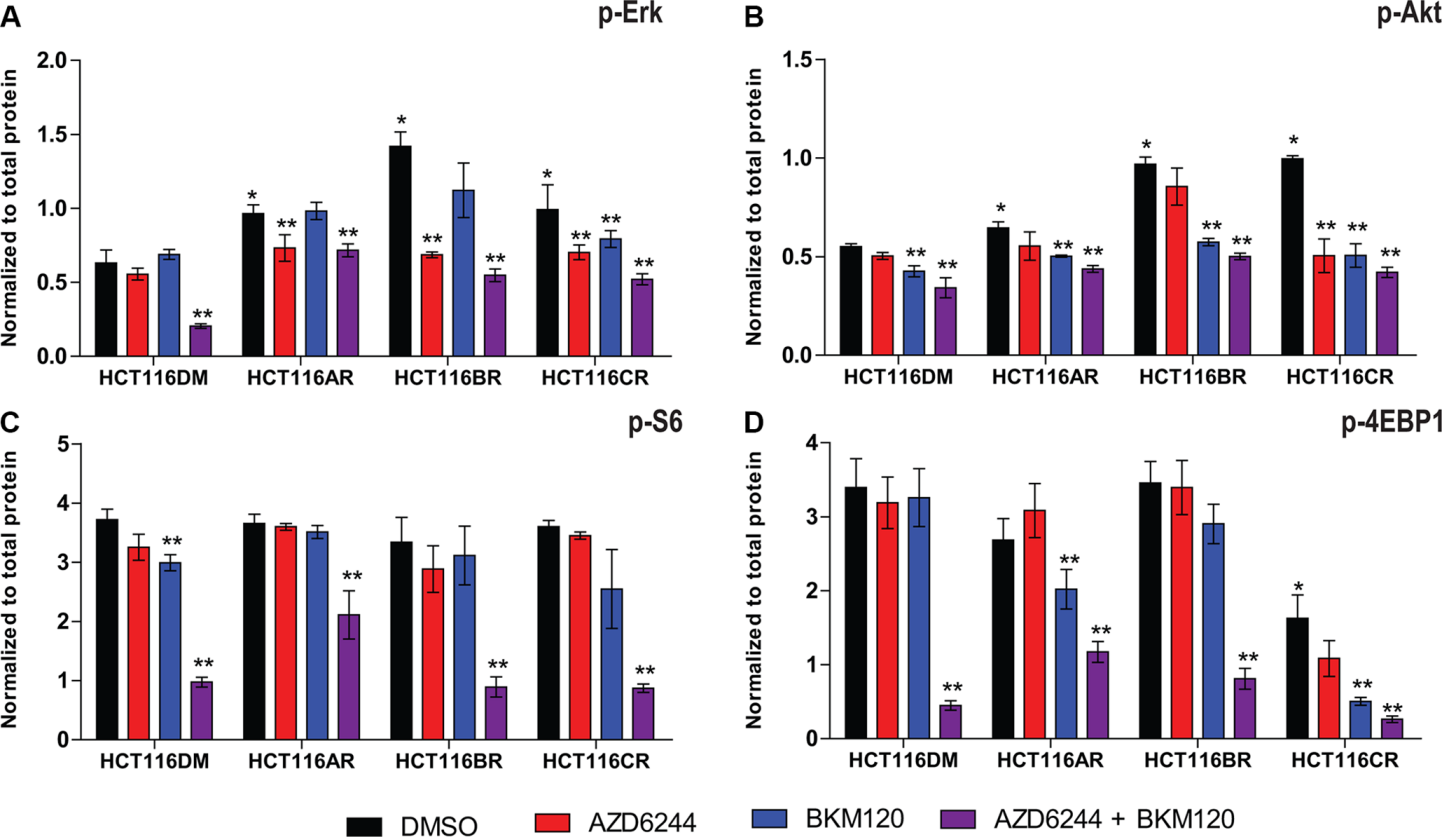
Pathway signaling levels of AQR cell lines Phosphorylation levels of (**A**) Erk, (**B**) Akt, (**C**) S6 and (**D**) 4EBP1 at 24 h post-treatment in HCT116DM, HCT116AR, HCT116BR and HCT116CR cells treated with vehicle (DMSO), AZD6244 alone (IC_50_ concentration), BKM120 alone (IC_50_ concentration), and their combination (IC_50_ + IC_50_ concentration). Levels were measured by ELISA. All experiments were repeated three times, and data are displayed as mean ± standard deviation of phosphorylated protein normalized to total protein. *indicates *p* < 0.05 compared to levels in HCT116DM. **indicates *p* < 0.05 compared to the control levels in the treated cell lines.

Analysis of baseline p-Erk, p-Akt, p-S6 and p-4EBP1 revealed HCT116AR cells had higher levels of p-Erk than HCT116DM cells (Figure 1), consistent with a previous report [11]. HCT116BR cells had elevated p-Erk and p-Akt. HCT116CR cells also had increased p-Erk and p-Akt, but also reduced p-4EBP1.

Following combination treatment, p-Erk, p-Akt, p-S6 and p-4EBP1 were reduced in all cells, indicating pathway inhibition activity was retained. AZD6244 treatment also reduced p-Erk in all cells, and BKM120 treatment reduced p-Akt in all cells, indicating that the inhibitory activity of single agents was retained as well. BKM120 also reduced p-4EBP1 in HCT116CR and HCT116AR but not HCT116BR cells, suggesting the AQR of HCT116BR cells to PI3K inhibition could involve reduced p-4EBP1 inhibition. AZD6244 also significantly reduced p-Akt, and p-4EBP1 (not statistically significant; *p* = 0.06) in HCT116CR but not the other cells.

### Cell phenotype analysis

**Figure 2:**
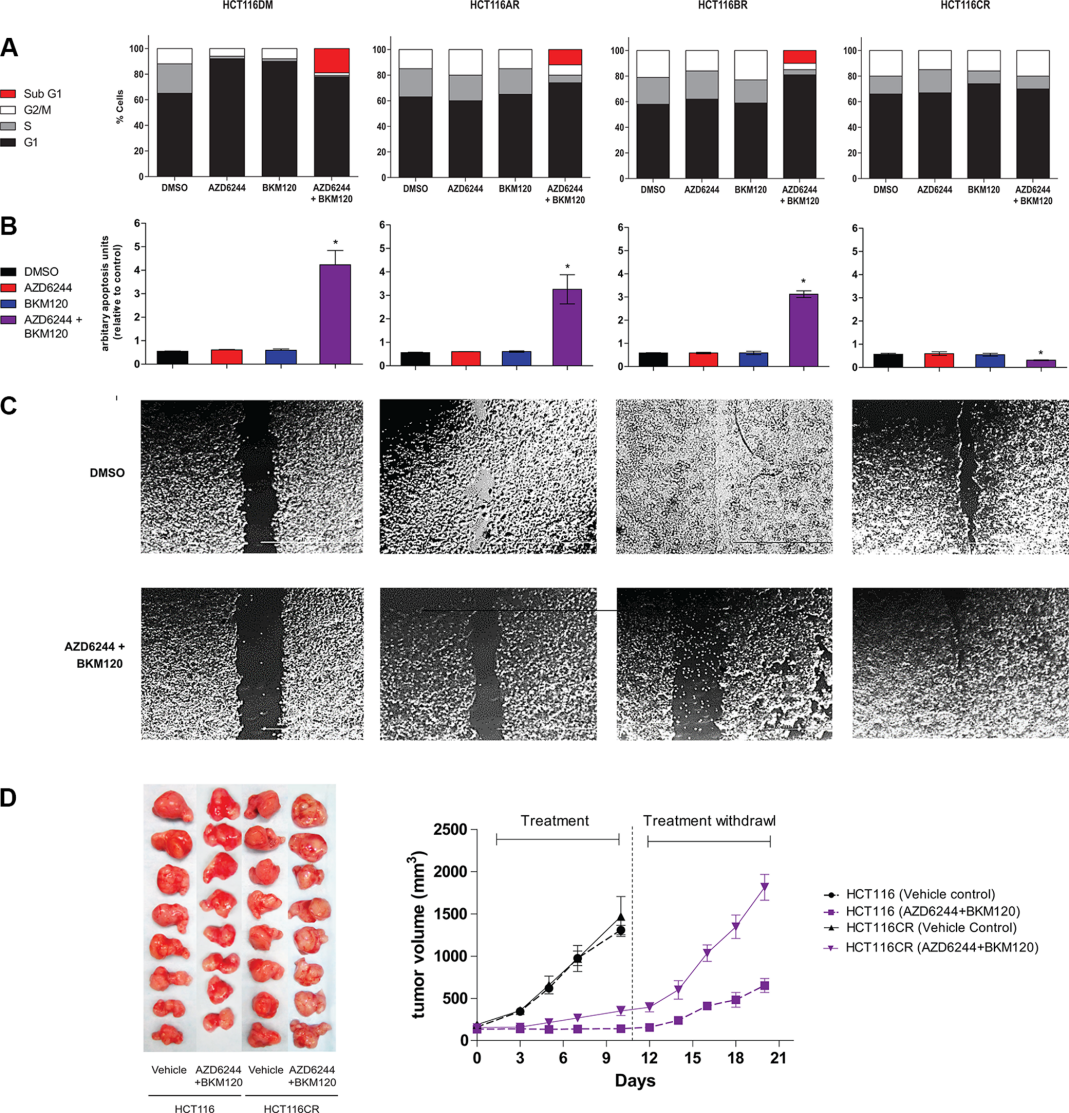
*In-vitro* and *in-vivo* phenotypes of AQR cell lines (**A**) Cell cycle phase distribution of cells at baseline and 24 h after exposure to IC_50_ concentrations of AZD6244 alone, BKM120 alone and AZD6244 and BKM120 combined. (**B**) Apoptosis of cells at baseline and 24 h after exposure to IC_50_ concentrations of AZD6244 alone, BKM120 alone and AZD6244 and BKM120 combined. *indicates *p* < 0.05 compared to cells treated with vehicle (DMSO). (**C**) Phase contrast images of cells in the wound-healing assay at baseline and 24 h after exposure to IC_50_ concentrations of AZD6244 alone, BKM120 alone and AZD6244 and BKM120 combined. (**D**) Images of tumors (left panel) and a chart of tumor volume over time (right panel) of tumors established from HCT116DM or HCT116CR cells in male SCID mice as described in online methods. All experiments were repeated three times, and data are displayed as mean ± standard deviation where relevant.

Consistent with their known activity [12, 13], single agent AZD6244 and BKM120 treatment led to increased G1 phase populations, and combination treatment led to an increased sub-G1 population and apoptosis in HCT116DM cells (Figure 2). In HCT116AR and HCT116BR cells, the increase in G1 phase populations was not observed following single agent treatment. However, combination treatment still led to an increased sub-G1 population and apoptosis in these cells. In HCT116CR cells, there was no significant increase in G1 or sub-G1 populations or apoptosis following single agent and combination treatment (Supplementary Figure S1). Indeed, apoptosis after combination treatment was significantly reduced in HCT116CR cells compared to HCT116DM cells. In wound healing experiments, HCT116AR, HCT116BR and HCT116CR cells displayed increased migration compared to HCT116DM cells when treated with DMSO. Combination treatment led to a reduction in migration of HCT116DM, HCT116AR and HCT116BR, but not HCT116CR cells.

### Drug cross-resistance analysis

No significant differences were observed in the sensitivity of HCT116-derived cells to 5-Fluorouracil, carboplatin and sorafenib, suggesting that a multi-drug resistance phenotype was not responsible for resistance (Supplementary Table S2). Consistent with the earlier observations, HCT116AR, HCT116BR and HCT116CR cells were more resistant than HCT116DM cells to an extended panel of MEK and PI3K inhibitors. Interestingly, HCT116CR cells also exhibited sensitivity to Akt and mTOR inhibitors. HCT116BR cells shared sensitivity with HCT116CR cells to Akt inhibitors. HCT116AR cells were more resistant to mTOR inhibitors.

### *In vivo* assessment

HCT116CR and HCT116DM cells were implanted in male *SCID* mice as described in Methods. Treatments started when the tumors reached the size of approximately 130–150 mm^3^, and consisted of either treatment with vehicle or the combination of AZD6244 (25 mg/kg) and BKM120 (40 mg/kg) daily. Treatment ceased after 10 days when the tumors in the mice treated with vehicle reached their maximum allowed tumor volume. Consistent with the treatment synergy observed *in-vitro*, HCT116DM tumor growth was significantly inhibited by treatment with the combination compared to vehicle (*p* = 0.01, Figure 2). Consistent with the AQR observed *in-vitro*, combination treatment reduced tumor growth less in HCT116CR than HCT116DM tumors, however the difference was not statistically significant. After treatment withdrawal, tumors from mice receiving combination treatment were measured for an additional 10 days. Tumors from mice implanted with HCT116CR cells grew at a faster rate than those implanted with HCT116DM cells (*p* = 0.03).

### DNA variant analysis

All 8 cells were screened for DNA variants in 50 prominent genes in cancer using the Ion AmpliSeq Cancer Hotspot Panel v2. The average depth of sequencing was 3,792 ± 424 reads and uniformity of base coverage was 98.53 ± 0.01% (Supplementary Table S3). All variants in HCT116 and LoVo cells that were expected to be detected according to previous reports [14] and assay design were detected. Four additional variants were detected: *SMAD4* (Y412H) in HCT116AR, *TP53* (T18A) and *TP53* (Y236C) in HCT116CR, and *PTEN* (A126S) in LoVoDM cells.

### RNA expression analysis

**Figure 3:**
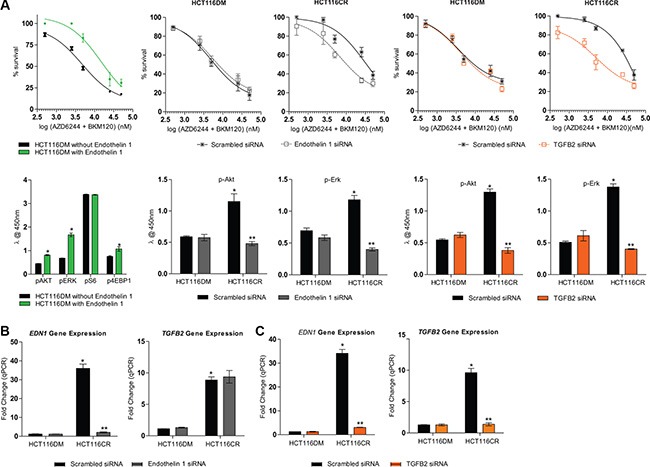
Effect of modulation of *EDN1* and *TGFB2* in HCT116DM and HCT116CR cells (**A**) Concentration response curves (top panel) and pathway signaling (bottom panel) of HCT116DM cells with or without extracellular supplementation of 100 nM endothelin-1 and treated with the combination of AZD6244 and BKM120 at a fixed-ratio of their IC_50_. (**B**) *EDN1* and *TGFB2* mRNA levels (top panel), Dose response curves of treatment with the combination of AZD6244 and BKM120 (middle panel) and levels of p-Akt and p-Erk (bottom panel) in HCT116DM and HCT116CR cells transfected with scrambled or *EDN1* siRNA. (**C**) *EDN1* and *TGFB2* mRNA levels (top panel), Dose response curves of treatment with the combination of AZD6244 and BKM120 (middle panel) and levels of p-Akt and p-Erk (bottom panel) in HCT116DM and HCT116CR cells transfected with scrambled or *TGFB2* siRNA. All experiments were repeated three times, and data are displayed as mean ± standard deviation. RNA expression was determined by real-time PCR, and normalized to ACTB levels and ratios in parental HCT116 cells. Protein phosphorylation levels were measured by ELISA, and normalized to total protein levels. *and **indicates *p* < 0.05 compared to HCT116DM and HCT116CR controls respectively.

Using gene expression arrays, eleven probes were differentially expressed (adjusted *p* < 0.01 and fold-change > 1.5) and consistent between HCT116CR and LoVoCR cells compared to the others (Supplementary Figure S2). Endothelin-1 (*EDN1)* and transforming growth factor beta 2 (*TGFB2*) had the highest differential expression (3.84 and 3.94 higher in CR cells respectively), of which the *p*-value was lowest for *EDN1* (1.22 × 10^−9^ and 2.44 × 10^−6^ respectively). The significant difference in *EDN1* and *TGFB2* expression between CR and DM cells was verified by real-time PCR (Figure 3B and 3C) and western immunoblotting in both HCT116- and LoVo-derived cells (Supplementary Figure S3). The increased expression of both *TGFB2* and *EDN1* was of particular interest given that *TGFB2* can upregulate *EDN1* through SMAD activation [15, 16]. No changes in the expression of EDN1 and TGFB2 were observed in the single agent AQR cells, and expression of endothelin receptors also remained unchanged in both the combination AQR cells compared to parental cells (Supplementary Figure S4).

### Modulation of *EDN1* and *TGFB2*

To investigate the involvement of EDN1 in drug resistance, parental HCT116 were cultured with 100 nM endothelin-1 or with vehicle for 24 hours before treatment with the combination of AZD6244 and BKM120. The two drugs were antagonistic in cells cultured with endothelin-1 (CI_fu0.5_ = 1.9 ± 0.05) while they were synergistic in HCT116 cells cultured with vehicle (0.19 ± 0.02). Cells cultured with endothelin-1 also had higher p-Akt, p-Erk and p-4EBP1 levels than those cultured with vehicle (Figure 3).

HCT116 and HCT116CR cells were also transfected with *EDN1* and *TGFB2* siRNA, and confirmed to have reduced *EDN1* and *TGFB2* RNA levels respectively compared to cells transfected with scramble siRNA. *TGFB2* siRNA transfection also reduced *EDN1* RNA levels, while *EDN1* siRNA transfection did not reduce *TGFB2* RNA levels, consistent with TGFB2 being a upstream regulator of *EDN1* [15, 16]. Transfection of *EDN1* siRNA in HCT116CR cells led to synergism between AZD6244 and BKM120 (0.52 ± 0.03), while antagonism remained in cells transfected with scrambled siRNA (2.1 ± 0.05). In parental HCT116 cells, the two drugs remained synergistic whether transfected with *EDN1* siRNA (0.25 ± 0.04) or scrambled siRNA (0.22 ± 0.02). Similarly, transfection with *TGFB2* siRNA (0.62 ± 0.08) but not scrambled siRNA (1.9 ± 0.07) also converted antagonism to synergism in HCT116CR cells. There was also no change in the synergism of combination treatment from the transfection of *TGFB2* siRNA (0.49 ± 0.06) or scramble siRNA (0.32 ± 0.05) in HCT116 cells. *EDN1* and *TGFB2* siRNA also both reduced the levels of p-Akt and p-Erk in HCT116CR cells, but not HCT116 cells. The same results were observed from endothelin-1 and siRNA priming of LoVo-derived cells (Supplementary Figure S5). Additionally, synergy between PI3K and MEK inhibitors was also restored in HCT116CR (CI@fu0.5 = 0.53 ± 0.02, *p* < 0.05) and LoVoCR (CI@fu0.5 = 0.61 ± 0.04, *p* < 0.05) cells in the presence of a fixed-low growth inhibitory concentration (5% growth inhibition) of bosentan, a dual endothelin receptor antagonist (Supplementary Figure S6).

## DISCUSSION

During the execution of this study, two controlled studies of AQR to combination treatment were reported. Ahronian *et al.* described AQR to combination BRAF and MEK inhibitor treatment in melanoma cells *in-vitro* that was associated with acquisition of *KRAS* amplification [17]. *KRAS* amplification was also observed in a tumor at recurrence in a melanoma patient treated with combination BRAF and MEK inhibitor therapy. Pirazolli *et al.* reported on AQR to combination afatinib and cetuximab treatment in *EGFR*-mutant lung cancer *in-vitro and in-vivo* that was linked to acquisition of mTOR activation [18]. Similar resistance and aberration was observed in a lung cancer patient treated with afatinib and cetuximab.

A major element in the consideration of combination therapy however, is the effect of the drug interaction, be it synergistic, additive or antagonistic [19]. Traditionally, this interaction has been measured by the protocol of Chou and Talalay [19]. The protocol allows calculation of a CI value derived from IC_50_ values of respective drugs at fixed ratios over a concentration range. Through this approach, the nature of interaction of combined drugs can be quantified, and misinterpretation of “one-sided” enhancement as synergy can be avoided.

The primary hypothesis of this study was that an altered drug interaction could have a role in AQR to combination therapy, based on the rationale that drug interaction can be a determinant of combination treatment efficacy. To test this hypothesis, models of AQR to either the combination of AZD6244 and BKM120 (HCT116CR), AZD6244 alone (HCT116AR), or BKM120 alone (HCT116BR) were generated through prolonged treatment, along with cells treated with DMSO vehicle (HCT116DM). In support of the hypothesis, CI values revealed the combination of AZD6244 and BKM120 was antagonistic in HCT116CR cells, while it remained synergistic in HCT116DM cells (Table 1). Combination treatment also remained synergistic in HCT116AR and HCT116BR cells, indicating that the loss of synergy was a specific feature of AQR in HCT116CR cells. In addition, pathway signaling analysis revealed that both single agent and combination treatment continued to inhibit expected targets in all cells. This suggested that factors other than loss of direct inhibitory activity were involved in the resistance.

Numerous measures were included in this study to support the validity of the loss of synergy. For single agent treatment, two doses of drugs at ½ IC_50_ concentrations were used to provide consistency with combination treatment in the number of drug aliquots. Cells with prolonged vehicle treatment were used to control for the effects of treatment over time. Cells were treated with alternative MEK (GDC0973) and PI3K (BYL719) inhibitors and similar trends were observed (Table 1), supporting that the observations were not compound specific. Parallel lines of LoVo cells were generated and similar patterns of resistance were observed in these cells (Supplementary Table S1), indicating that the observations were also not cell line specific. Lastly, tumor xenografts confirmed the resistance phenotype *in-vivo* (Figure 2).

Many other differences between AQR to combination and single agent treatment were revealed by the study design. Phosphorylation of 4EBP1 was uniquely reduced, and AZD6244 treatment reduced p-Akt and p-4EBP1 in HCT116CR cells but not others (Figure 1). In addition to reduced G1 arrest observed in HCT116AR, HCT116BR and HCT116CR cells, apoptosis was reduced in HCT116CR cells following combination treatment (Figure 2). Cell migration was less impeded by combination treatment in HCT116CR cells compared to all other cells. While HCT116AR cells were more resistant to mTOR inhibitors, HCT116CR cells were more sensitive. These additional phenotypes caution against the assumption that resistance to combination treatment is a composite of resistance to its component agents.

To gain insight into potential mechanisms for the loss of synergy, sequencing of 50 genes frequently mutated in cancer was performed. Unique DNA variants were detected in *SMAD4 (Y412H)* in HCT116AR, *PTEN (A126S)* in LoVoAR, and *TP53 (T18A)* and *TP53 (Y236C)* in HCT116CR cells. All these variants were predicted to be “deleterious” and “probably damaging” by SIFT [20] and Polyphen [21]. The *PTEN (A126S)* variant partially inactivates PTEN [22], and thereby could activate PI3K signaling and compensate for MEK pathway inhibition. Inactivation of p53 by *TP53 (18A)* and *TP53 (Y236C)* mutation could be involved in AQR, given the role of p53 in tumor suppression and stress response [23]. Nonetheless, these aberrations were not consistent between models, prompting further investigation.

Gene expression array analysis revealed *EDN1* and *TGFB2* as the top candidate genes overexpressed in both HCT116CR and LoVoCR cells but not in the other cells (Supplementary Figure S2). The *EDN1* gene encodes for endothelin-1, a vasoactive peptide, which is typically associated with vasoconstriction and endothelial functions [24]. Endothelin-1 binds to endothelin receptors A and B, which are G-protein coupled receptors (GPCRs), and via second messengers activate many signaling pathways, including MEK and PI3K pathways [24]. Endothelin-1 can also trigger activation of protein kinase C [25], which can lead directly to Erk and Akt activation [26, 27]. TGFβ is a well-known cytokine that mediates numerous processes such as proliferation, differentiation, survival, adhesion, and migration through its activation of the SMAD family of proteins [28]. Interestingly, inhibition of MEK and PI3K signaling can lead to TGFβ activation [29], and *EDN1* is a known downstream target of TGFβ/SMAD activated transcription [15, 16].

**Figure 4:**
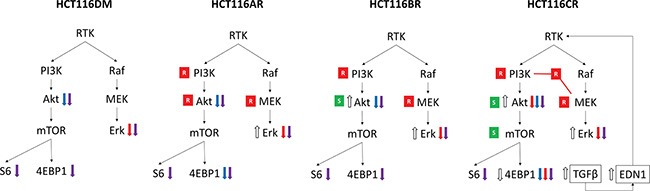
Overview of differences in phenotypes of AQR cells An increase (⇑) or decrease (⇓) in phosphorylation levels at baseline is indicated by white arrows. An increase (⇑) or decrease (⇓) in phosphorylation levels post-treatment are indicated by colored arrows, according to treatment with AZD6244 alone (red), BKM120 alone (blue) and their combination (purple). Resistance (red box with white R) and sensitivity (green box with white S) to inhibitors of respective proteins in drug sensitivity analysis are also indicated. Also indicated is the increased expression of *EDN1* and *TGFB2* that was observed uniquely in HCT116CR cells, and its hypothesized activation of pathway signaling.

Taken together, a mechanism through which HCT116CR and LoVoCR cells AQR to combination treatment can be rationalized (Figure 4). It can be hypothesized that the combined inhibition of PI3K and MEK pathways led to the activation of TGFβ and subsequently *EDN1*. The increased endothelin-1 then provided compensatory activation of the PI3K and MEK pathway, contributing to resistance to combination treatment. In support of this hypothesis, supplementation with endothelin-1 converted the synergism of combination treatment in parental HCT116 and LoVo cells to antagonism, along with activating Akt, Erk and 4EBP1 (Figure 3, Supplementary Figure S5). Silencing of *EDN1* or *TGFB2* by siRNA also changed the antagonism of combination treatment in HCT116CR and LoVoCR cells to synergism, and reduced p-Akt and p-Erk levels. Additionally, bosentan, a dual endothelin receptor antagonist restored synergy between BKM120 and AZD6244 in HCT116CR and LoVoCR cells (Supplementary Figure S6).

However, it is less clear how a change in drug interaction has occurred. Historically, synergy or antagonism could be understood as an enhancement or reduction of receptor binding or enzyme activity resulting from the combination of two agents [30]. Without a common receptor or enzyme in this study, one approach could be to consider the coordinated inhibition of the MEK and PI3K pathways and its downstream effects as the singular target of drug interaction. With this consideration, the upstream activation of MEK and PI3K pathways by endothelin-1 through GPCRs [31], or activation of Erk and Akt downstream of MEK and PI3K inhibition [5], can be viewed as means to decouple pathway inhibition and/or bypass inhibitor effects, and thereby alter the singular system. It also remains that endothelin-1 could activate alternative pathways that modify the downstream events of MEK and PI3K inhibition through its multiplicity of pathways. The reduced apoptosis and inhibition of migration of HCT116CR cells following combination treatment (Figure 2) could be a manifestation of this effect. The unexpected sensitivity of HCT116CR cells to Akt and mTOR inhibitors could be indicative of altered pathway dynamics, while also highlighting a potential rationale for intervention.

In conclusion, the results of this study have supported the hypothesis that an altered drug interaction can determine AQR to combination treatment. Differences in the phenotypes of AQR to single agent and combination treatment have indicated that caution should be exercised in assuming mechanisms of resistance for combination treatment can be determined from studies of single agent treatment. Overexpression of the *TGFB2-EDN1* axis was identified as a potential mechanism of acquired loss of synergy for combination MEK and PI3K inhibitor therapy, and it would be of interest to observe whether this mechanism has a role in clinical resistance as current trials of this combination mature. A precise delineation of the mechanisms of altered drug interaction could not be achieved, as the protocol of Chou and Talalay only informs on the overall nature of interaction and not its components [19]. Systems biology characterization of the complex multiplicity of effectors and pathways in the *TGFB2-EDN1* axis may help to shed further light on how drug interactions can affect drug resistance in its modern setting.

## MATERIALS AND METHODS

### Generation of cells with acquired resistance

HCT116 and LoVo colorectal cancer cells were obtained from American Type Culture Collection (Manassas, VA). Cells were cultured in DMEM and supplemented with 10% fetal bovine serum (Invitrogen, Carlsbad, CA), 50,000 units penicillin and 50 mg streptomycin (Sigma, St Louis, MO) at 37°C in a humidified atmosphere containing 5% CO_2_. Cell lines were continuously exposed to either a) two aliquots of vehicle (DMSO), b) two aliquots of ½ IC_50_ concentration of AZD6244, c) two aliquots of ½ IC_50_ concentration of BKM120, or d) aliquots of IC_50_ concentration of AZD6244 and BKM120 each for a period of 3–8 months. The growth of the cells was monitored weekly and acquisition of resistance was indicated by normal proliferation of cells under the described selection pressures. The cells were maintained with the relevant concentrations of compounds or DMSO respectively. Clonal selection was not carried out for these various AQR cell lines. All the parental and resistant cells passed the authentication testing using short tandem repeat profiling performed by Promega GenePrint^®^ 10 system (Madison, WI, USA). The cell lines were tested at the commencement and also at the completion of the study.

### Drug sensitivity analysis

AZD6244, BKM120, BYL719, GDC0973, sorafenib, trametinib, BEZ235, Ku-0063794, RAD001, MK2206 and bosentan were obtained from Selleck Chemicals (Houston, TX). Carboplatin and 5-FU were obtained from Sigma. All stock solutions were prepared in DMSO (MP Biomedicals, Solon, OH) at a final concentration in culture media of 0.25% (v/v). Cells in 90 μl medium were seeded (3000 cells/well) onto 96-well microtitre plates (Nunc, Rochester, NY). After 24 hours, 10 μl of medium containing compounds in graded concentrations ranging from 0.1 μM to 1000 μM was added to the wells. Control wells contained 20 μl of relevant solvent to achieve a final concentration of 0.25% of each solvent. The effect on cell numbers was assessed using the CellTiter 96^®^ AQueous Non-Radioactive Cell Proliferation Assay (Promega, Madison, WI) (MTS Assay) at 72 h post-treatment. The IC_50_ was calculated as the drug concentration that inhibited cell proliferation by 50% compared to vehicle controls as previously described [32].

### Drug combination analysis

The effect of combining compounds was evaluated using the median-effect equation and combination index (CI) method of Chou and Talalay [10]. For fixed-ratio experiments the concentrations of each compound to reduce the absorbance to 50% of that obtained with control cells (inhibitory concentration 50%; IC_50_) were generated. Most experiments were performed by combining both agents added together at a fixed 1:1 ratio of the IC_50_ of each individual drug or a fixed low growth inhibitory dose. The effects of the combination were calculated for each experimental condition using an spreadsheet based on the median-effect analysis method of Chou and Talalay [10]. We have previously described more details of this analysis [33]. For each level of fraction unaffected (fu), a CI was calculated as follows: CI = (*D*)_1_/(*D*_f_)_1_ + (*D*)_2_/(*D*_f_)_2_ + [(*D*)_1_ (*D*_f_)_2_/(*D*_f_)_1_ (*D*_f_)_2_], where (*D*)_1_ and (*D*)_2_ are the concentrations of the combination required to produce fu, and (*D*_f_)_1_ and (*D*_f_)_2_ are the concentrations of the individual drugs required to produce fu. Data giving linear regression coefficients (*r*^2^) of median-effect plots < 0.95 were excluded. CI values of < 1, 1 and > 1 were considered to indicate synergy, additivity and antagonism respectively. CI values with the non-exclusive assumption have been reported.

### ELISA analysis

Levels of p-AKT (Ser473), p-mTOR (Ser2448), p-S6 (Ser235/236), p-4eBP1 (Thr37/46) and their respective total proteins were measured using PathScan^®^ Sandwich ELISA kits (Cell Signaling Technology, Beverly, MA) on the Infinite 200 Pro (Tecan, Mannedorf, Switzerland) according to the manufacturer's instructions.

### Cell cycle analysis

Exponentially growing cells were seeded into 25 cm^2^ tissue culture flasks at 1 × 10^6^ cells/flask and allowed to attach for 24 h prior to drug addition. Following 24 h incubation, both attached and detached cells were collected and fixed with 2 ml of ice-cold 70% ethanol. Following centrifugation at 900 *g* for 5 mins, the pellet was resuspended in 800 μl of PBS containing 100 μl of 1 mg/mL RNaseA and 100 μl of 400 μg/mL propidium iodide (both from Sigma) and stored overnight at 4°C. Samples were analysed on a LSRII flow cytometer (BD, Franklin Lanes, NJ) equipped with an argon laser tuned to 488 nm and the red fluorescence collected at 630 nm. The data was analysed using WinMDI v 2.8 and DNA histograms were gated on a display of DNA peak signal against DNA area to exclude debris and clumps.

### Apoptosis measurement

Apoptosis was measured using the Cell Death ELISA^®^ (Roche, Mannheim, Germany) kit. Cells were plated in 96-well plates (3000 cells/well) and on the following day treated with drug or solvent in a volume adjusted to 200 μL with 10% FCS/DMEM. After 24 hours, nucleosomes were quantified according to the manufacturer's instructions.

### *In vivo* assessment

The study received ethics board approval at the National Cancer Centre of Singapore and Singapore General Hospital. All mice were maintained according to the “Guide for the Care and Use of Laboratory Animals” published by National Institute of Health, USA. They were provided with sterilised food and water *ad libitum*, and housed in negative pressure isolators with 12 h light/dark cycles. HCT116 parental and HCT116CR cells were implanted in both flanks of male *SCID* mice aged 9–10 weeks. Each injection consisted of approximately 5 × 10^6^ cells. Treatments started when the tumors reached the size of approximately 150–200 mm^3^. Mice bearing tumors were treated as follows: AZD6244 (25 mg/kg) + BKM120 (40 mg/kg) *p.o.* daily, and vehicle controls receiving PEG300/captisol (30:30:water) *p.o.* daily. Each treatment arm involved 4 independent tumor-bearing mice. The treatment lasted for 10 days. Bi-dimensional measurements were performed twice a week and tumor volumes are calculated based on the following formula: Tumor volume = [(Length) × (Width^2^) × (p/6)]. Tumors in the vehicle-treated groups were harvested on day 10 when the tumor size reached ~ 1500 mm^3^. Tumors in AZD6244/BKM120 group were allowed to grow for an additional 10 days before harvesting. The data were plotted as means and standard errors for each treatment group versus time. At the end of the study, the mice were sacrificed and tumor samples collected.

### Mutational analysis

DNA was extracted from cells using the DNA easy Blood and Tissue kit (Qiagen, Hilden, Germany) and quantified using the Picogeren method (Thermo Fisher, Waltham, MA). Mutation analysis by next generation sequencing was performed using the Ion AmpliSeq Cancer Hotspot Panel v2 (Thermo Fisher) on the Ion Torrent PGM instrument (Thermo Fisher) according to the manufacturer's instruction. This panel consists of 207 amplicons covering approximately 22 kb of regions in 50 genes with known cancer associations. A total of 2 μl DNA were used to generate barcoded libraries using the IonXpress barcode adapters (Thermo Fisher). Amplified libraries were quantified using Tapestation High Sensitivity D1000 screentape (Agilent Technologies, Santa Clara, CA) and up to 16 barcoded libraries were combined to a final concentration of 10 pM. The pooled libraries underwent amplification by emulsion PCR on Ion Spheres Particles and enrichment using the Ion One Touch System (Thermo Fisher). Sequencing was performed on the Ion Torrent PGM with the 318 chip (Thermo Fisher). Reads were aligned to hg19 and variant called using Ion Torrent Suite 3.6.2 (Thermo Fisher). Variants were annotated using Ensembl Variant Effect Predictor v75 and filtered for variants that were frequent in less than 5% of the Asian population in the 1000 genomes project, and non-synonymous.

### Gene expression analysis

Total RNA was isolated using the RNeasy Mini kit (Qiagen) and confirmed for quality using the RNA 6000 Nano kit on the Agilent Bioanalyzer 2100 (Agilent Technologies). Gene expression analysis was performed using Affymetrix Human Gene 1.0ST genechips on the Affymetrix Fluidics Station 450 (Affymetrix, Santa Clara, CA). From 200 ng of total RNA, cDNA was generated, fragmented, and biotin labelled using the Applause WT-Amp ST system (NuGEN, San Carlos, CA). The prepared targets were hybridized overnight to the arrays, after which the arrays were washed, stained and scanned according to manufacturer's instructions. Affymetrix CEL files were normalized by the Robust Multi-array Average (RMA) method using the R/bioconductor Affy library. We used the TM4-MeV software to perform both the unsupervised hierarchical clustering and differential gene expression via the LIMMA module.

### Extracellular endothelin-1 priming

Exponentially growing HCT116 or LoVo parental cells were seeded in 96 well plates (3000 cells/well). After 24 h, the culture medium was replaced by serum-free medium for an additional 24 h in the presence or absence of 100 nM endothelin-1 (Sigma). After 24 h of priming, cells were washed with sterile PBS and re-introduced to normal serum containing medium and combination drug exposure carried out as described in drug sensitivity analysis.

### siRNA treatment

Two independent EDN1 (Cat # SR301329) and TGFB2 (Cat #SR304807) siRNA were obtained from Origene Technologies (Rockville, MD). Cells were transfected with siRNA at concentrations of 10 nM and exposed to a range of concentrations of AZD6244, BKM120 or their combination 24 h post-transfection. The effect on cell numbers was measured at 72 h post-transfection by the CellTiter 96^®^ AQueous Non-Radioactive Cell Proliferation Assay (Promega). Validation of knockdown efficiency was performed on the ABI7900HT real time system (Thermo Fisher) using Taqman gene expression assay for *EDN1*, *TGFB2* and *ACTB*. Relative quantities were measured by the ΔΔCT method [34], using *ACTB* as a reference gene and parental HCT116 cells as a calibrator.

### Statistical analysis

Differences in IC_50_ values and protein ELISA were assessed using a paired sample *t*-test. A one-sample *t*-test (two-tailed) was used to compare the CI_fu0.5_ with the predicted value for additivity of 1. For apoptosis, one-way ANOVA followed by Dunn's Multiple Comparison test was performed to assess for differences. All statistical analyses were two-tailed and performed using GraphPad Prism 5.00 software (GraphPad Software Inc., San Diego, CA). Statistical significance was considered when *p* < 0.05.

## SUPPLEMENTARY MATERIALS FIGURES AND TABLES


