# Supplementary material for: Acquired resistance to combination treatment through loss of synergy with MEK and PI3K inhibitors in colorectal cancer
Source: Oncotarget. 2016 Apr 11;7(20):29187–98. doi: 10.18632/oncotarget.8692 (PMC5045388; doi:10.18632/oncotarget.8692)
Supplement: Supplementary file 1 [file oncotarget-07-29187-s001.pdf]

Acquired resistance to combination treatment through loss of synergy with MEK and PI3K inhibitors in colorectal cancer

Supplementary Materials

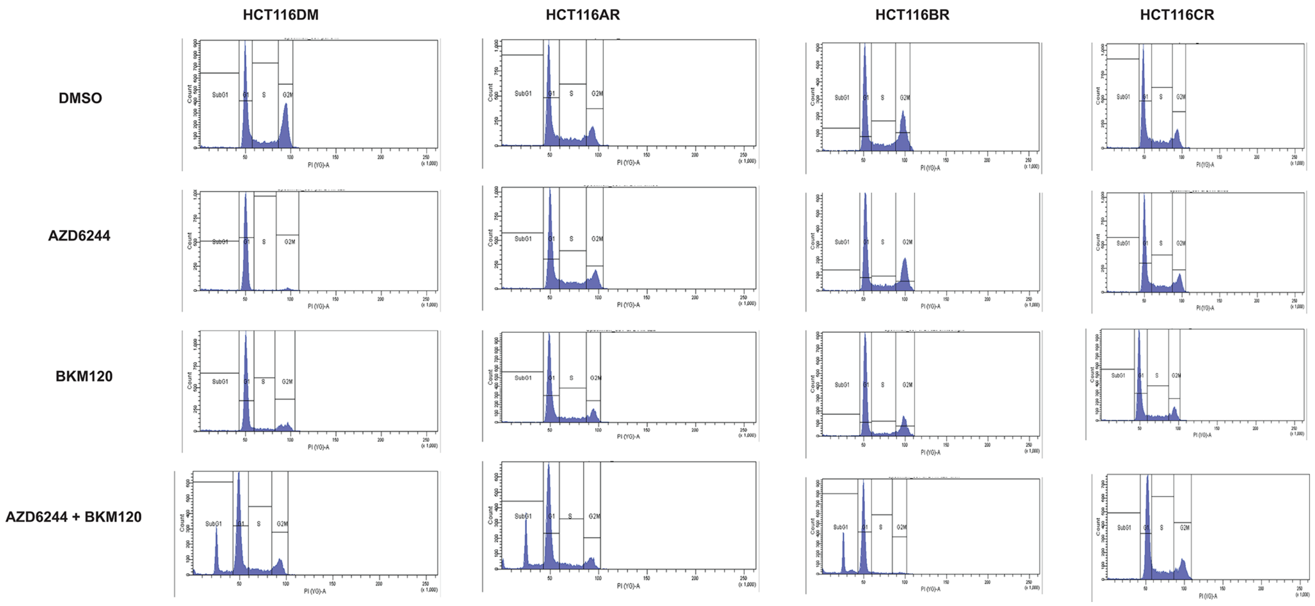

Supplementary Figure S1: Histograms showing cell cycle profiles of HCT16DM, HCT116AR, HCT116BR and HCT116CR cells exposed to DMSO, IC_{50} concentrations of AZD6244 or BKM120 and combination of AZD6244 and BKM120 (IC_{50} + IC_{50}).

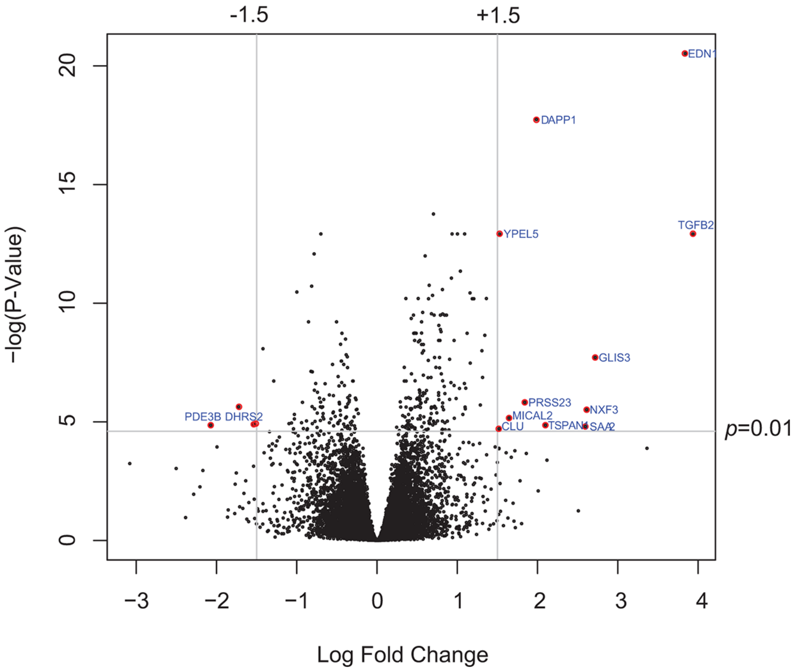

Supplementary Figure S2: Volcano plot of genes according to their fold change in expression and *p*-value of differences between CR and DM cells of HCT116 and LoVo in gene expression array analysis. Guides (dashed lines) for fold differences of 1.5 and *p*-values of 0.01 are indicated on the chart.

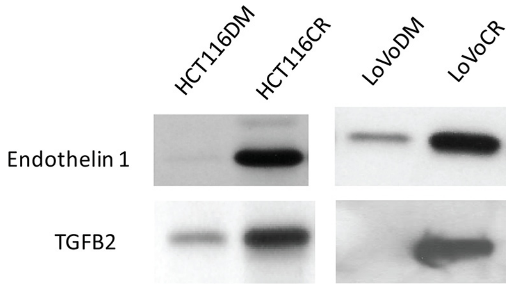

Supplementary Figure S3: Protein levels of endothelin 1 and TGFB2 in HCT116DM, HCT116CR, LoVODM and LoVoCR cells. A Representative immunoblot of three independent experiments is displayed.

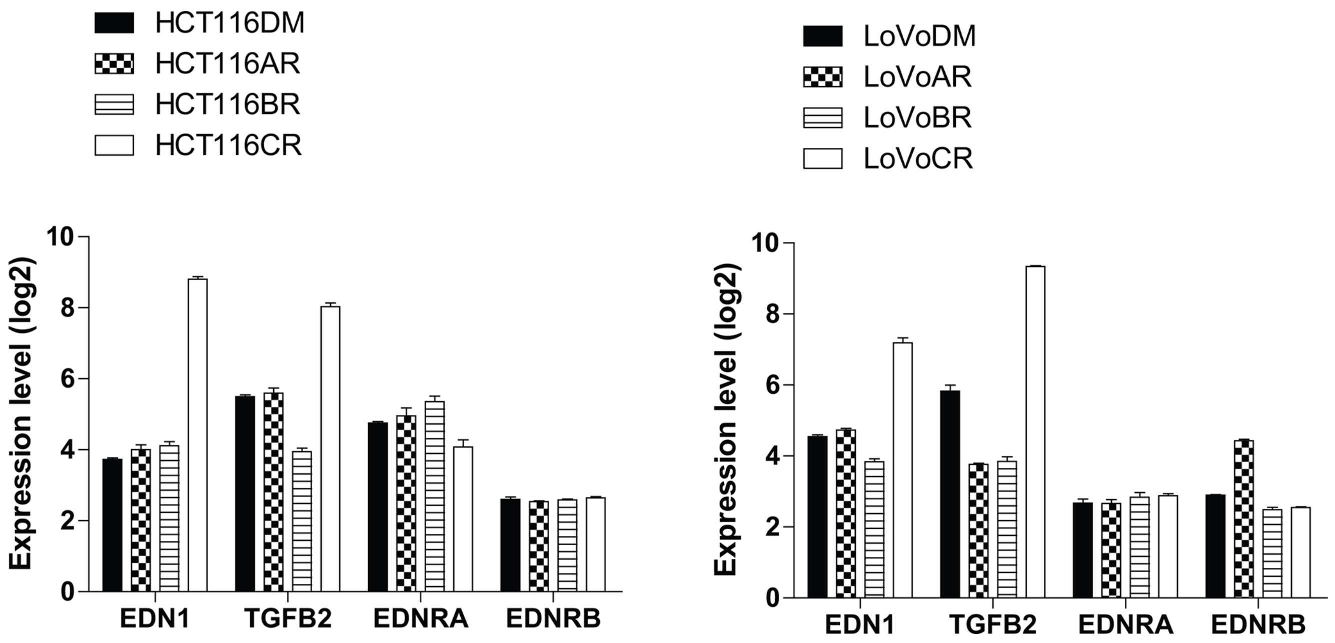

Supplementary Figure S4: Bar charts showing mRNA expression of EDN1, TGFB2, EDNRA and EDNRB in all three replicates of HCT116 or LoVo-derived DM, AR, BR and CR cells. Data are expressed as mean ± std. deviation.

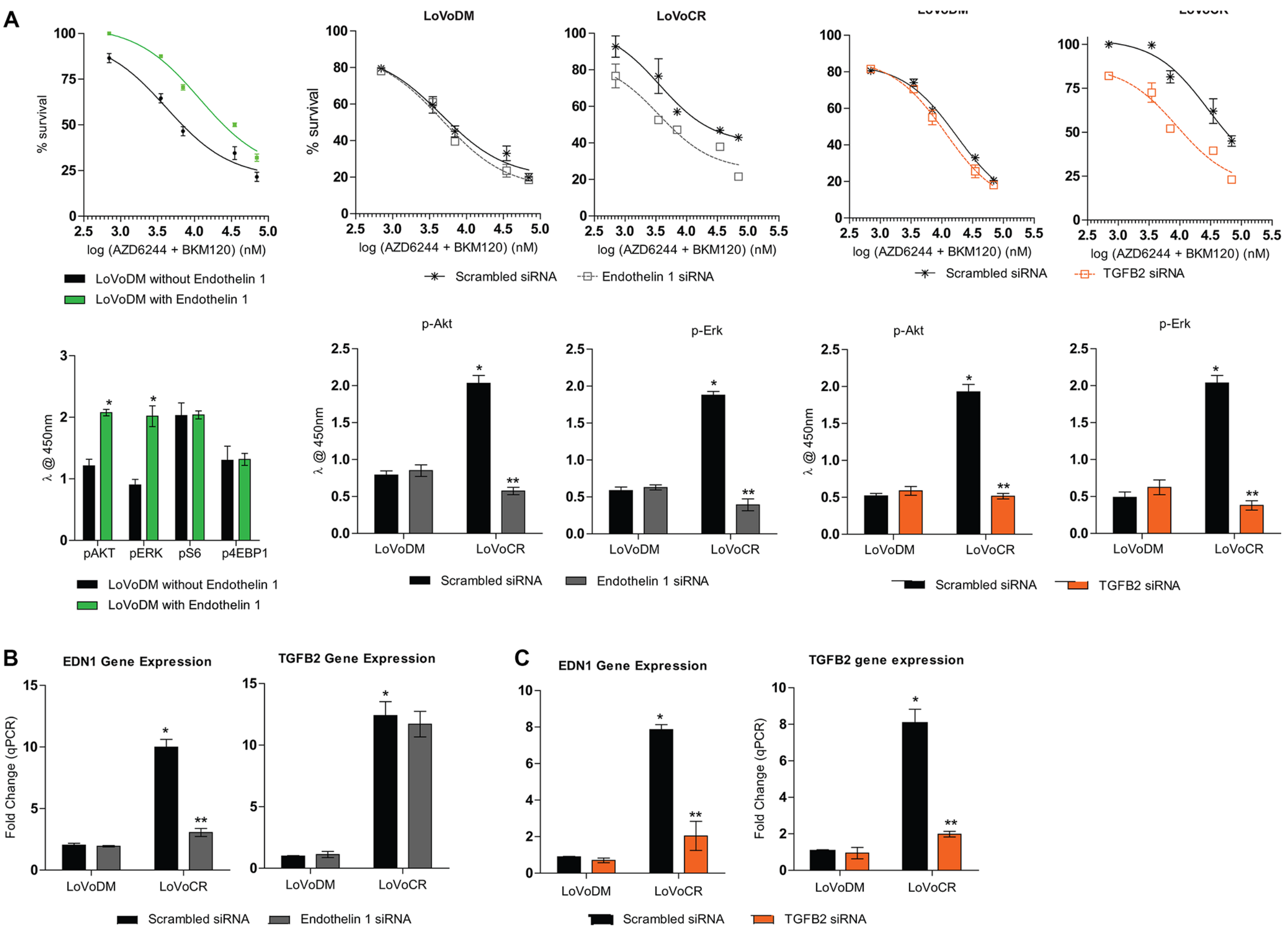

Supplementary Figure S5: Effect of modulation of *EDN1* and *TGFB2* in LoVoDM and LoVoCR cells. (A) Concentration response curves (top panel) and pathway signaling (bottom panel) of LoVoDM cells with or without extracellular supplementation of 100 nM endothelin-1 and treated with the combination of AZD6244 and BKM120. **(B)** *EDN1* and *TGFB2* mRNA levels (top panel), Dose response curves of treatment with the combination of AZD6244 and BKM120 (middle panel) and levels of p-Akt and p-Erk (bottom panel) in LoVoDM and LoVoCR cells transfected with scrambled or *EDN1* siRNA. **(C)** *EDN1* and *TGFB2* mRNA levels (top panel), Dose response curves of treatment with the combination of AZD6244 and BKM120 (middle panel) and levels of p-Akt and p-Erk (bottom panel) in LoVoDM and LoVoCR cells transfected with scrambled or *TGFB2* siRNA. All experiments were repeated three times, and data are displayed as mean ± standard deviation. RNA expression was determined by real-time PCR, and normalized to ACTB levels and ratios in parental LoVo cells. Protein phosphorylation levels were measured by ELISA, and normalized to total protein levels. *and **indicates $p < 0.05$ compared to LoVoDM and LoVoCR controls respectively.

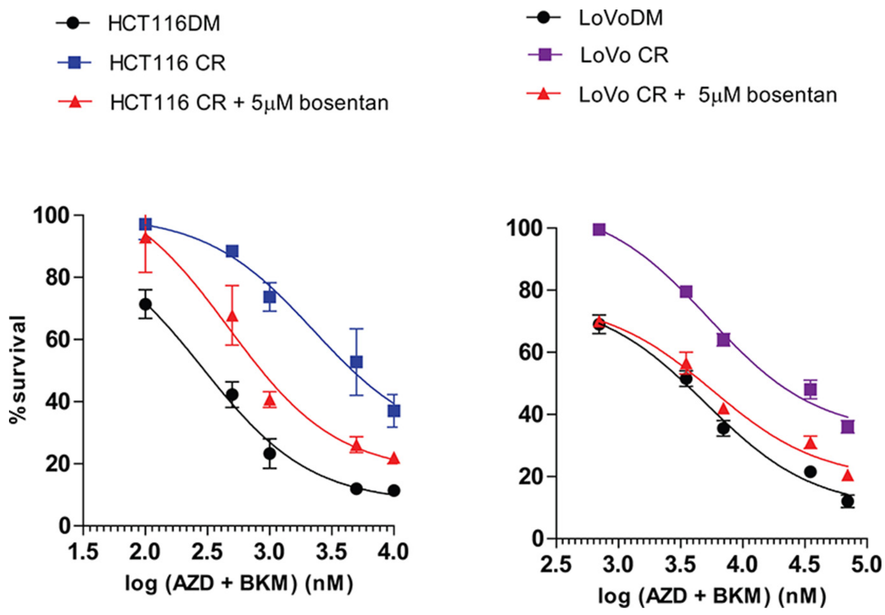

Supplementary Figure S6: Dose response curves showing effect of low-growth inhibitory concentration of bosentan on combination of AZD6244 and BKM120 in HCT116CR and LoVoCR cells. Also shown are the dose response curves of AZD6244 and BKM120 combination in HCT116DM and LoVoDM cells as positive controls.

Supplementary Table S1: IC_{50} and combination index values of treatment with various drugs and their combinations in LoVo-derived cells

Treatment	LoVoDM	LoVoAR	LoVoBR	LoVoCR
AZD6244 IC_{50} (μM)	8.9 ± 1.1	31.2 ± 2.5*	18.4 ± 1.9*	21.2 ± 2.2*
BKM120 IC_{50} (μM)	1.2 ± 0.2	14.1 ± 1.8*	6.5 ± 0.8*	8.3 ± 1.1*
AZD6244 + BKM120 IC_{50} (μM)	0.9 ± 0.11	1.3 ± 0.2	1.1 ± 0.1	10.2 ± 1.4*
AZD6244 + BKM120 $CI_{fu0.5}$	0.35 ± 0.02	0.41 ± 0.06	0.34 ± 0.02	2.72 ± 0.08*
GDC0973 IC_{50} (μM)	6.8 ± 1.24	23.1 ± 3.5*	14.1 ± 2.2*	9.7 ± 1.9*
BYL719 IC_{50} (μM)	0.9 ± 0.01	8.2 ± 0.8*	5.4 ± 0.9*	4.7 ± 0.7*
GDC0973 + BYL719 IC_{50} (μM)	0.8 ± 0.02	0.9 ± 0.4	1.0 ± 0.04	6.8 ± 0.6*
GDC0973 + BYL719 $CI_{fu0.5}$	0.29 ± 0.01	0.25 ± 0.05	0.36 ± 0.01	3.21 ± 0.12*

The IC_{50} values of AZD6244, BKM120, GDC0973, and BYL719 as single agents and in combination (in the presence of the other drug at fixed ratio of their IC_{50} values) are indicated. CI values for fraction unaffected at IC_{50} ($fu_{0.5}$) are also given. Additivity = 1, Antagonism > 1, Synergy < 1.
*$p < 0.05$ for differences in IC_{50} values compared to LoVoDM, and for differences to 1 for CI values.

Supplementary Table S2: IC_{50} values (µM) of treatment with various agents in respective HCT116-derived cells

Compound	Target	HCT116DM	HCT116AR	HCT116BR	HCT116CR
5-Fluorouracil	Cytotoxic	8.4 ± 0.4	7.1 ± 0.2	10 ± 0.3	11 ± 1.0
Carboplatin	Cytotoxic	4.7 ± 0.9	3.9 ± 0.3	5.2 ± 0.4	4.6 ± 0.6
Sorafenib	Pan Kinase	7.6 ± 0.6	13.5 ± 0.9	9.6 ± 0.6	6.7 ± 0.7
Trametenib	MEK	4.7 ± 0.7	56.6 ± 4.9*	48 ± 4.1*	6.0 ± 0.3*
GDC0973	MEK	5.6 ± 0.5	20.6 ± 1.3*	21.1 ± 2.1*	18.3 ± 1.1*
BYL719	PI3K	10 ± 0.2	31.2 ± 2.6*	39.5 ± 3.5*	25.5 ± 2.1*
GDC0941	PI3K	> 100	> 100	> 100	> 100
BEZ235	PI3K/mTOR	9.2 ± 0.3	67.5 ± 3.8*	11.2 ± 0.6	1.9 ± 0.3*
Ku-0063794	mTOR	8.7 ± 0.4	61.9 ± 3.9*	10 ± 0.4	3.9 ± 0.4*
RAD001	mTOR	> 100	> 100	> 100	> 100
MK2206	AKT	20.5 ± 0.8	16.4 ± 1.1	9.4 ± 0.9*	1.1 ± 0.2*

Supplementary Table S3: Next generation sequencing output and variants detected in respective cells

Sample ID	HCT116DM	HCT116AR	HCT116BR	HCT116CR
Number of mapped reads	722,034	760,328	780,150	851,690
Percent base reads on target	98.31%	98.55%	98.42%	98.33%
Average base coverage depth	3,300	3,480	3,601	3,909
Uniformity of base coverage	98.67%	99.39%	98.18%	96.20%
Variants, total number	25	24	24	26
Variants, filtered*	ABL1_Y276C (40.8%)	ABL1_Y276C (36.7%)	ABL1_Y276C (43.9%)	ABL1_Y276C (39.7%)
	CTNNB1_Ser45del (42.4%)	CTNNB1_Ser45del (38.1%)	CTNNB1_Ser45del (42.2%)	CTNNB1_Ser45del (41.3%)
	KRAS_G13D (52.1%)	KRAS_G13D (73.8%)	KRAS_G13D (93.6%)	KRAS_G13D (95.3%)
	PIK3CA_H1047R (49.1%)	PIK3CA_H1047R (50.5%)	PIK3CA_H1047R (55.4%)	PIK3CA_H1047R (51.2%)
	SMO_V404M (49.6%)	SMO_V404M (66.4%)	SMO_V404M (48.9%)	SMO_V404M (65.9%)
		SMAD4_Y412H (48.3%)		TP53_T18A (49.8%)
				TP53_Y236C (51.7%)
**Sample ID**	**LoVoDM**	**LoVoAR**	**LoVoBR**	**LoVoCR**
Number of mapped reads	871,915	762,193	1,021,235	862,241
Percent base reads on target	98.02%	98.07%	98.01%	98.12%
Average base coverage depth	3,975	3,487	4,640	3,946
Uniformity of base coverage	98.60%	99.38%	98.74%	99.06%
Variants, total number	20	18	20	19
Variants, filtered*	APC_R1114* (35.6%)	APC_R1114* (49.9%)	APC_R1114* (39.7%)	APC_R1114* (49.2%)
	APC_M1431CfsTer42 (65.8%)	APC_M1431CfsTer42 (50.8%)	APC_M1431CfsTer42 (59.2%)	APC_M1431CfsTer42 (50.5%)
	FBXW7_R505C (50.8%)	FBXW7_R505C (50.9%)	FBXW7_R505C (49.1%)	FBXW7_R505C (49.9%)
	KRAS_G13D (63.6%)	KRAS_G13D (63.3%)	KRAS_G13D (68.4%)	KRAS_G13D (65.3%)
	SMO_A324T (31.9%)	SMO_A324T (32.2%)	SMO_A324T (32.8%)	SMO_A324T (31.9%)
	PTEN_A126S (6%)			

*Number in parenthesis indicates the variant allele frequency from sequencing.
